# Glucocorticoids Directly Affect Hyaluronan Production of Orbital Fibroblasts; A Potential Pleiotropic Effect in Graves’ Orbitopathy

**DOI:** 10.3390/molecules28010015

**Published:** 2022-12-20

**Authors:** Erika Galgoczi, Monika Katko, Fruzsina Reka Papp, Robert Csiki, Sara Csiha, Annamaria Erdei, Miklos Bodor, Bernadett Ujhelyi, Zita Steiber, Ferenc Gyory, Endre V. Nagy

**Affiliations:** 1 Division of Endocrinology, Department of Internal Medicine, Faculty of Medicine, University of Debrecen, Nagyerdei Krt. 98, 4032 Debrecen, Hungary; 2 Doctoral School of Health Sciences, University of Debrecen, Nagyerdei Krt. 98, 4032 Debrecen, Hungary; 3 Department of Ophthalmology, Faculty of Medicine, University of Debrecen, Nagyerdei Krt. 98, 4032 Debrecen, Hungary; 4 Department of Surgery, Faculty of Medicine, University of Debrecen, Nagyerdei Krt. 98, 4032 Debrecen, Hungary

**Keywords:** Graves’ orbitopathy, hyaluronan, glucocorticoids, hyaluronan synthase, methylprednisolone, prednisolone, hydrocortisone, dexamethasone

## Abstract

Orbital connective tissue expansion is a hallmark of Graves’ orbitopathy (GO). In moderate-to-severe active GO, glucocorticoids (GC) are the first line of treatment. Here we show that hydrocortisone (HC), prednisolone (P), methylprednisolone (MP), and dexamethasone (DEX) inhibit the hyaluronan (HA) production of orbital (OF) and dermal (DF) fibroblasts. HA production of GO OFs (*n* = 4), NON-GO OFs (*n* = 4) and DFs (*n* = 4) was measured by ELISA. mRNA expression of enzymes of HA metabolism and fibroblast proliferation was examined by RT-PCR and BrdU incorporation, respectively. After 24 h of GC treatment (1µM) HA production decreased by an average of 67.9 ± 3.11% (*p* < 0.0001) in all cell cultures. *HAS2*, *HAS3* and *HYAL1* expression in OFs also decreased (*p* = 0.009, *p* = 0.0005 and *p* = 0.015, respectively). Ten ng/mL PDGF-BB increased HA production and fibroblast proliferation in all cell lines (*p* < 0.0001); GC treatment remained effective and reduced HA production under PDGF-BB-stimulated conditions (*p* < 0.0001). MP and DEX reduced (*p* < 0.001, *p* = 0.002, respectively) PDGF-BB-induced *HAS2* expression in OFs. MP and DEX treatment decreased PDGF-BB stimulated *HAS3* expression (*p* = 0.035 and *p* = 0.029, respectively). None of the GCs tested reduced the PDGF-BB stimulated proliferation rate. Our results confirm that GCs directly reduce the HA production of OFs, which may contribute to the beneficial effect of GCs in GO.

## 1. Introduction

Graves’ orbitopathy (GO) is an autoimmune disease, which is the most common extrathyroidal manifestation of Graves’ disease (GD) [1]. GO is characterized by increased orbital connective tissue volume and thickening of the external eye muscles. Major symptoms are conjunctival redness, upper eyelid retraction, periorbital oedema, double vision, increased tearing, photosensitivity, exophthalmos, and pain in the orbit [1,2]. GO has a major impact on the quality of life [3]. All management guidelines, including the most recent ones [4] suggest the administration of intravenous (i.v.) glucocorticoids (GCs) for moderate-to-severe, active GO as first-line treatment. The effectiveness of GCs is generally linked to their immunosuppressive effect (reviewed in [5]). These include anti-inflammatory [6] and immunosuppressive actions via inhibiting lymphocytic infiltration of the tissues [5,7]. The pathogenesis of GO includes infiltration of immune cells, inflammation, increased proliferation rate of fibroblasts, and accumulation of hyaluronan (HA) [8,9]. HA is able to hold up to a thousand times more water than its own weight, resulting in the oedematous swelling of the orbital connective tissues and raising the pressure inside boundaries of the bony orbit [10]. The HA cycle is maintained by the hyaluronan synthase enzymes (HAS1, HAS2, and HAS3) and hyaluronidases (HYAL1 and HYAL2) [11].

The initial phase of the pathogenesis of GO is characterized by the increased activity of Th1 lymphocytes, promoting the production of IL-1β, IL-2, TNF-α and IFN-γ and platelet derived growth factor (PDGF) BB [1,12]. PDGF-BB shows increased expression in the orbital tissues, and is known to stimulate fibroblast proliferation and production of HA [12]. In the present study, we aimed to test the effect of different GCs on the proliferation and HA production of orbital fibroblasts (OF), and mRNA expression of HA synthases and hyaluronidases under basal and PDGF-BB stimulated conditions.

## 2. Results

**Figure 1:**
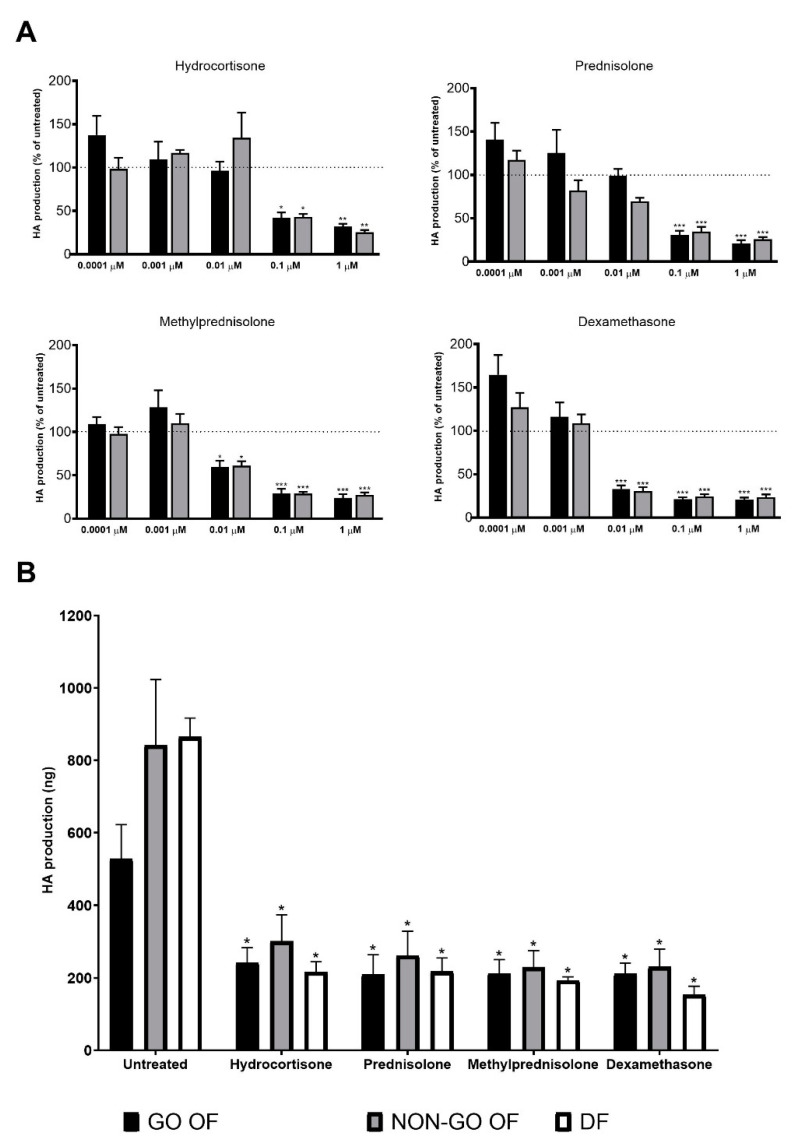
(**A**) The dose-dependent effect of 0.0001–1.0 µM GCs on hyaluronan (HA) production, compared to untreated cells * *p* < 0.01, ** *p* < 0.001, *** *p* < 0.0001. (**B**) The influence of GCs (1 µM) on HA production compared to untreated cultures. Data were analyzed using a repeated measures ANOVA followed by Dunnett’s test. Results shown are the mean ± SEM. * *p* < 0.0001. HA—hyaluronan, GO OF—Graves’ orbitopathy fibroblasts, NON-GO OF—control orbital fibroblasts, DF—dermal fibroblasts.

The lowest concentrations of the different GCs with maximal inhibitory effect on HA production of OFs were tested in the range of 0.0001–1.0 µM concentration (Figure 1A). Basal HA production of GO and NON-GO OFs was not different (*p* = 0.168). Both MP and DEX exerted a significant decreasing effect on the HA secretion in 0.01 µM concentration. In contrast, the inhibitory effects of P and HC were observed at 0.1 µM. As the maximal inhibitory effect of all GCs in all fibroblast lines tested was present at 1.0 µM concentration, for further experiments this concentration was selected for all GCs.

Basic HA production of OFs and DFs did not differ (*p* = 0.974). GC treatment markedly decreased HA production (*p* < 0.0001) and the degree of inhibition did not depend on the origin of the cells (*p* = 0.066) (Figure 1B). Regardless of the baseline HA concentration, all GCs were effective (*p* < 0.0001).

**Figure 2:**
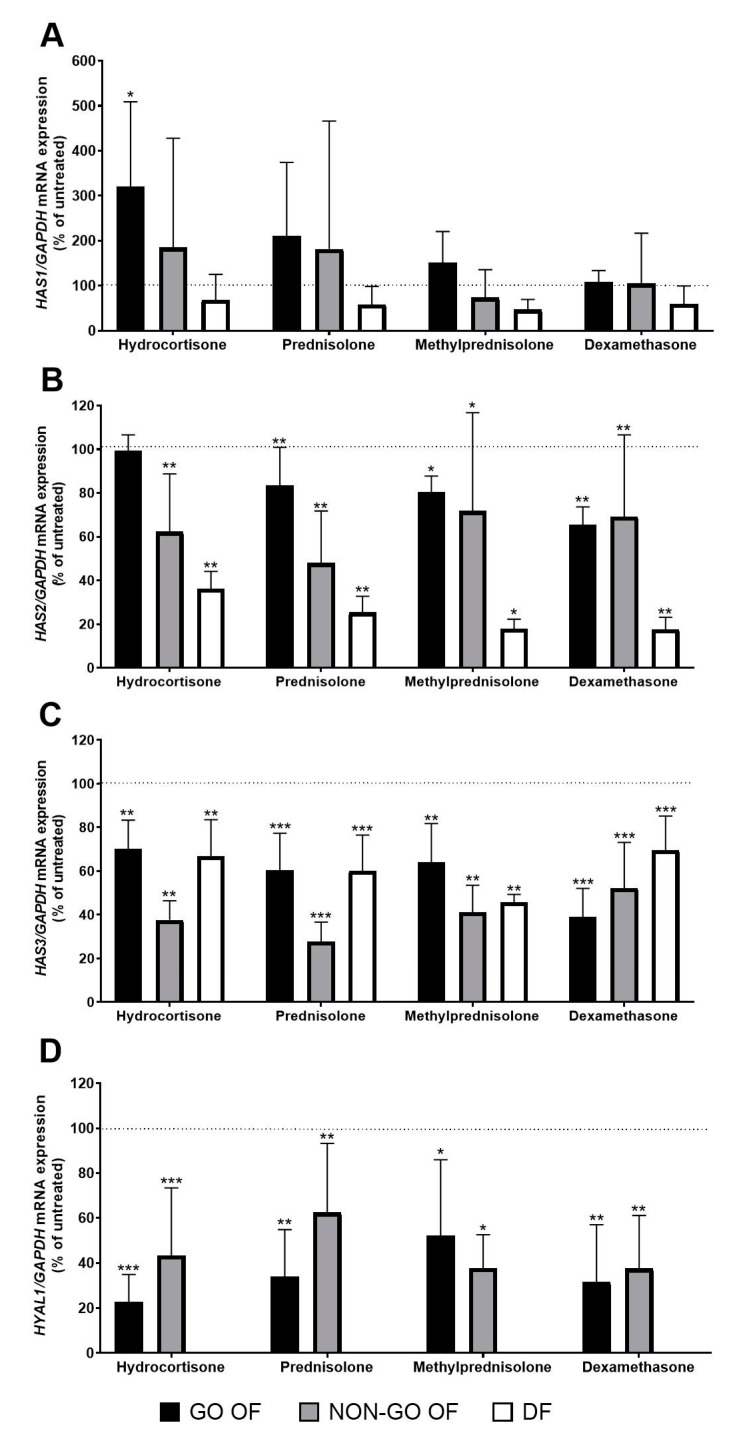
The effect of GCs (1 µM) on (**A**) *HAS1*, (**B**) *HAS2*, (**C**) *HAS3* and (**D**) *HYAL1* mRNA expression of OFs and DFs. Data were analyzed using a repeated measure ANOVA followed by Dunnett’s test. The dotted line at 100 % represents the mRNA expression of untreated cultures. Results shown are the mean ± SEM. * *p* < 0.05, ** *p* < 0.01, *** *p* < 0.001. GO OF—Graves’ orbitopathy fibroblasts, NON-GO OF—control orbital fibroblasts, DF—dermal fibroblasts, *HAS1*—hyaluronan synthase 1, *HAS2*—hyaluronan synthase 2, *HAS3*—hyaluronan synthase 3, *GAPDH*—glyceraldehyde 3-phosphate dehydrogenase, *HYAL1*—hyaluronidase 1.

The *HAS1* expression of fibroblasts was not affected by the origin of the cells (GO vs. NON-GO: *p* = 0.795, OFs vs. DFs: *p* = 0.153), and the effect of GC treatment was significant in OFs (*p* = 0.037). Although *HAS1* expression of OFs had a tendency to be increased under the effect of GCs, the Dunnett’s test showed that HC was the only GC that caused a significant increase in *HAS1* expression of GO OFs (*p* = 0.026) (Figure 2A).

The expression of *HAS2* (*p* = 0.884) and the effect of GC treatment on that were not affected by the origin (GO vs. NON-GO) of the OFs (*p* = 0.236). GC treatment significantly reduced *HAS2* mRNA levels in OFs (*p* = 0.009) (Figure 2B). The basic expression of *HAS2* was higher in DFs compared to OFs (*p* = 0.005). The Dunnett’s tests showed that *HAS2* expression was reduced by all types of GC used (*p* < 0.0001), except for HC in GO OFs (*p* = 0.134).

Expression of *HAS3* did not depend on whether the cells originated from a GO or NON-GO orbital connective tissue (*p* = 0.848), and GC treatment had a reducing effect (*p* = 0.0005). *HAS3* expression of DFs and OFs did not differ (*p* = 0.473). According to the Dunnett’s test, each GC reduced *HAS3* expression in all cases, regardless of origin (HC: *p* < 0.0005, P: *p* < 0.0001, MP: *p* < 0.0005, DEX: *p* < 0.0001) (Figure 2C).

*HYAL1* mRNA expression was too low in DF cultures to accurately measure its basal and post-treatment expression. In OFs, basal *HYAL1* expression was not different in GO and NON-GO OFs (*p* = 0.956). GCs resulted in reduced expression levels in OFs (*p* = 0.015); Dunnett’s tests showed that *HYAL1* mRNA decreased in response to all types of GCs tested (HC *p* < 0.0007, P *p* < 0.0062, MP *p* < 0.05, DEX *p* < 0.002).

The basal *HYAL2* expression of GO OFs, NON-GO OFs and DFs was not different. GCs affected neither OFs nor DFs (*p* = 0.525). The lack of effect was independent from the origin of cells (*p* = 0.446).

**Figure 3:**
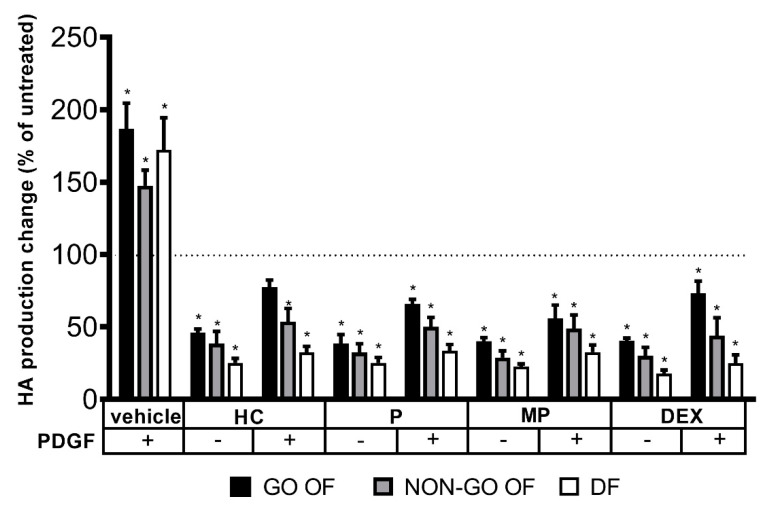
The influence of GCs (1 µM) in the absence and presence of PDGF-BB on hyaluronan (HA) production. The dotted line represents the HA production of untreated cells. Data were analyzed using a repeated measures ANOVA followed by Dunnett’s test. Result shown are mean ± SEM. * *p* < 0.0001. GO OF—Graves’ orbitopathy fibroblasts, NON-GO OF—control orbital fibroblasts, DF—dermal fibroblasts, HA—hyaluronan, PDGF—platelet derived growth factor BB, HC—hydrocortisone, P—prednisolone, MP—methylprednisolone, DEX—dexamethasone.

The origin of the cells did not affect the response to PDGF-BB treatment (GO vs. NON-GO: *p* = 0.593, DFs vs. OFs: *p* = 0.995), and PDGF-BB increased HA production in all cultures (*p* < 0.0001). GC treatment reduced HA production even under PDGF-BB-stimulated conditions (*p* < 0.0001), and the effect was cell-origin-dependent (*p* = 0.004); it was more intense in DFs. As shown in Figure 3, all types of GC were able to decrease the PDGF-BB-stimulated HA production below the basal HA production of the cells (*p* < 0.0001).

To investigate if GCs may alter the mRNA expression of HA synthases in the presence of PDGF-BB, we examined the cultures in which the 24 h 10 ng/mL PDGF-BB treatment increased HA synthase expression. PDGF-BB increased *HAS1* mRNA levels in three of four GO OFs, three of four NON-GO OFs and three of four DFs (*p* = 0.022) to a comparable extent (*p* = 0.381). In OFs, GCs had no effect on PDGF-BB-stimulated *HAS1* expression (GO: *p* = 0.300, NON-GO: *p* = 0.391). In contrast, in DFs GCs significantly reduced the stimulated *HAS1* expression (*p* = 0.048).

**Figure 4:**
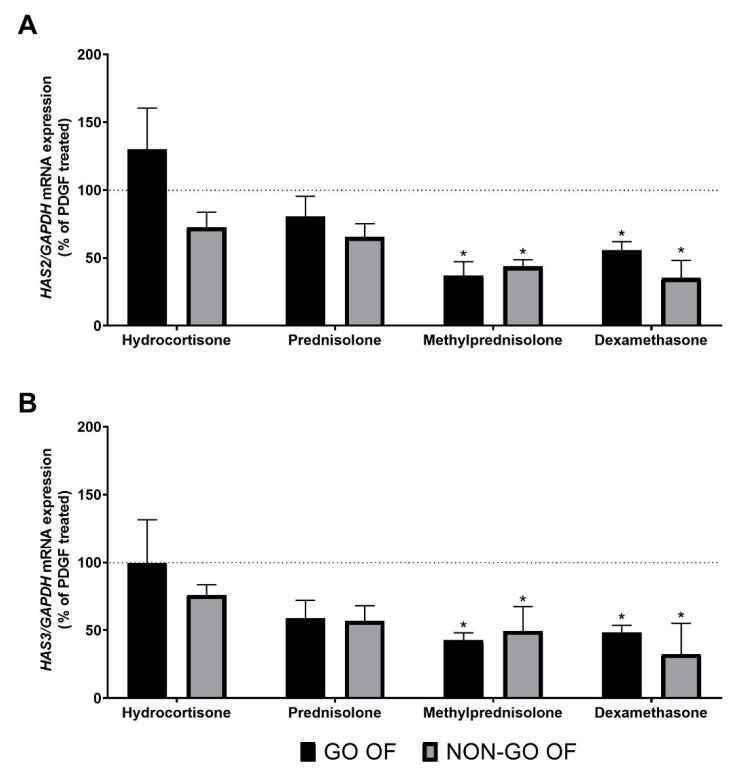
The effect of GCs (1 µM) on (**A**) *HAS2* and (**B**) *HAS3* mRNA expression after PDGF-BB treatment. The dotted line represents the mRNA expression of PDGF-BB stimulated cultures. Data were analyzed using a repeated measure ANOVA followed by Dunnett’s test. Result shown are the mean ± SEM. * *p* < 0.05. GO OF—Graves’ orbitopathy fibroblasts, NON-GO OF—control orbital fibroblasts, DF—dermal fibroblasts, *HAS2*—hyaluronan synthase 2, *HAS3*—hyaluronan synthase 3, *GAPDH*—glyceraldehyde 3-phosphate dehydrogenase.

PDGF-BB treatment increased *HAS2* expression in OFs (*p* = 0.003) regardless of the origin of the cells (GO vs. NON-GO: *p* = 0.297). The Dunnett’s test showed that only MP and DEX were able to reduce (*p* < 0.001, *p* < 0.002, respectively) PDGF-BB-induced *HAS2* expression, while HC and P were not (*p* = 0.997, *p* = 0.151, respectively) (Figure 4A). In DFs, the effect of PDGF-BB on *HAS2* expression was negligible (*p* = 0.772).

PDGF-BB enhanced (*p* = 0.049) *HAS3* mRNA expression in OFs (three out of four in GO, three out of four in NON-GO) (Figure 4B). The behavior of GO and NON-GO OFs under the treatment was not different (*p* = 0.723). According to Dunnett’s test, only MP and DEX treatment could decrease the PDGF-BB-induced increase in *HAS3* expression (*p* = 0.035 and *p* = 0.029, respectively). The post hoc test showed that MP (*p* = 0.044) and DEX (*p* = 0.045) were able to reduce the *HAS3* expression even below the baseline. PDGF-BB had no effect on *HAS3* expression in DFs (*p* = 0.535).

*HYAL1* mRNA expression decreased after PDGF-BB treatment (*p* < 0.003), in both GO and NON-GO OFs (*p* = 0.207). GCs had no effect on the reduced *HYAL1* expression caused by PDGF-BB (*p* = 0.366). Neither PDGF-BB treatment alone nor in combination with GC influenced *HYAL2* expression.

**Figure 5:**
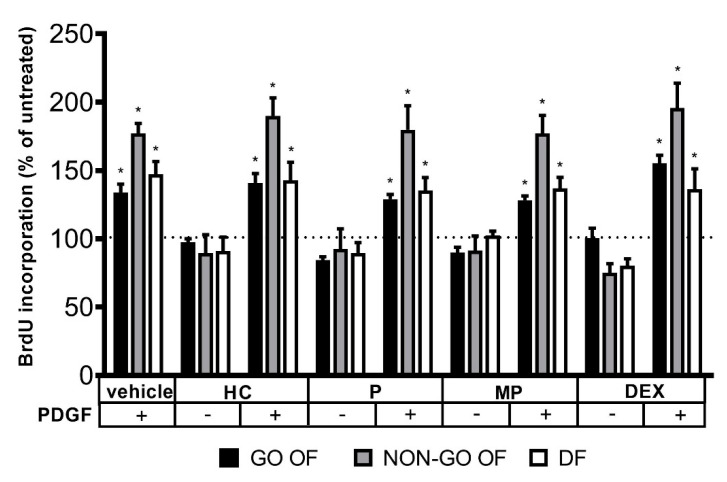
The proliferation rate of PDGF-BB treated cells. The dotted line represents the basal proliferation rate. Data were analyzed using repeated measure ANOVA followed by Dunnett’s test. Result shown are the mean ± SEM. * *p* < 0.0001. GO OF—Graves’ orbitopathy fibroblasts, NON-GO OF—control orbital fibroblasts, DF—dermal fibroblasts, BrdU—5-bromo-2’-deoxyuridine, PDGF—platelet derived growth factor BB, HC—hydrocortisone, P—prednisolone, MP—methylprednisolone, DEX—dexamethasone.

PDGF-BB increased the proliferation rate of every cell line (*p* < 0.0001). None of the GCs studied was able to prevent the PDGF-BB-stimulated proliferation of OFs in 1 µM concentration (Figure 5).

## 3. Discussion

The inflammatory mediators produced by infiltrating T cells and activated fibroblasts present in the orbit (e.g., TNF-α, IFN-γ, TGF-β, IL-1β, IGF-1, and PDGF) lead to increased glycosaminoglycan (GAG) synthesis of orbital fibroblasts; thus they contribute to the remodeling of the orbital tissue, which has a pivotal role in the pathogenesis of GO. [13,14]. The overwhelming component of GAG in cultured OFs is HA [8,9]. In this study, we confirmed that GCs, including MP, which is the first-line drug in the treatment of active GO [4], in addition to their known immunosuppressive effects, act directly on HA production and the mRNA expression of HA synthases in human OFs, even when stimulated with PDGF-BB.

It is known that GCs can inhibit T-cell-activating cytokines and adhesion molecules, as well as proliferation and infiltration of the immune cells [5]. On the other hand, GCs inhibit HA production of different cell types, e.g., DEX in human dermal fibroblasts [15,16,17], HC and DEX in cultured fibroblast-like synoviocytes and in leukocytes isolated from synovial fluid [18], as well as in thyrocytes and thyroid fibroblasts [19]. It has been shown that DEX inhibits GAG production of human OFs [20]. Our study aimed to explore the direct effect of different types of GCs on HA production and proliferation of human OFs; indeed, after a 24 h incubation with HC, P, MP or DEX, the production of HA substantially decreased in all cell types (Figure 1).

HA synthases are known to be influenced by GCs. In human DFs and in keratinocyte-derived immortalized cells, *HAS1* expression was very low to measure, and the *HAS2* expression was downregulated, while the *HAS3* expression did not change after 150 nM DEX treatment [15]. MP treatment reduced the mRNA expression of *HAS1* and *HAS2* in rats’ hearts before transplantation [21]. In fibroblast-like synoviocytes HC and DEX reduced the expression of all HAS enzymes [18]. Kaback et al. found using Northern analysis of OFs that DEX completely blocked the mRNA expression of *HAS* enzymes at 10 nM in an individual with severe GO [22]. Our results show that GCs act mainly by reducing *HAS2* and *HAS3* expression in OFs, which may explain the decrease in HA production. HAS2 is the most abundantly expressed HA synthase in OFs [23]. The HAS3 enzyme is more active than HAS1 and HAS2, in addition, its expression is associated with early macrophage-induced inflammation [24], which may suggest its important role in GO.

Currently there are little data on how GCs act on hyaluronidases. Gebhardt and colleagues found that, after DEX treatment HA production was reduced in human skin, but the expression and activity of *HYAL1* and *HYAL2* remained unchanged [15]. In contrast, in airway smooth muscle cells, GC treatment resulted in decreased *HYAL1* expression while it did not alter *HYAL2* expression [25]. Others found a decrease in *HYAL1* expression in mouse macrophages after DEX treatment [26]. These results are further supported by our observation that GCs decrease *HYAL1* expression. We assume that the decrease in HA production observed by us after GC treatment is the result of the combined effect of changes seen in *HAS2* and *HAS3* expressions; the reduction in HA degradation via decreased *HYAL1* expression may have a less marked effect on the overall process.

Although HYAL1 and HYAL2 are known to be the major enzymes of HA catabolism, there are newly described hyaluronidases, TMEM2 and CEMIP [27,28,29], whose role has not been investigated yet in OFs. However, the exact role of HA-degrading enzymes and the consequent fragmentation of HA in the pathogenesis of GO is still unclear and needs further studies.

In our experiments, the GCs did not change the proliferation rate of fibroblast cultures, except the DEX in DFs, suggesting that GCs did not act via reducing the proliferation of fibroblasts in the orbit.

PDGF-BB, a known stimulator for HA synthesis [12,30,31] increased HA production and proliferation in all cell cultures tested, irrespective of the site of the origin. We found that all studied GCs were able to decrease the HA production even in the presence of PDGF-BB. GCs not only prevented the stimulating effect of PDGF-BB, but reduced HA secretion further below baseline. GCs could alter the stimulatory effects of PDGF-BB in various ways. In vivo, GCs act as immunosuppressive agents, inhibiting the activation and infiltration of T cells into tissues [15,25]. The direct interaction between the glucocorticoid receptor (GR) and NF-κB may be responsible for the blocking effect of GCs on the NF-κB pathway [5,25,26]. Moreover, this linkage decreases the PDGF-BB expression [31,32]. Not only does the synthesis of PDGF-BB depend on NF-κB but it exerts its activating and stimulating effect through this signaling pathway [33], which may explain how GCs could diminish the PDGF-BB-stimulated effect on HA production. The promoter region of *HAS3* is known to carry a binding site for the transcription factor NF-κB [24]. The GC/GR dimer directly blocks NF-κB signaling pathway [34]; this may underlie the found effect.

One limitation of our study is the 24 h incubation time used in our experimental protocol, which may result in the disappearance of notable short-term changes at the transcriptional level. Further, we examined human primary cell cultures, and individual differences may have been reflected in mRNA expression levels of HAS enzymes and their response to treatments; only cultures that had responded to PDGF-BB treatment with increase were included in the analysis.

It is known that the actual expression levels of HAS enzymes do not always reflect HA production [35], which may explain seemingly contradictory results in some cases between HA production and HAS expression.

In general, PDGF-BB had a stimulatory effect on at least one of the HAS enzymes’ expression in tested cells. We found, in accordance with previous results [20,23], that OFs and DFs have different HAS mRNA expression patterns and also differ in their response to PDGF-BB, which had no effect on the expression of *HAS2* and *HAS3* in DFs, while it had an increasing effect in certain OF cultures. Each GC alone did decrease *HAS2* mRNA expression, while PDGF-BB alone increased it. After stimulation with PDGF-BB, GCs reduced the upregulated *HAS2* expression in fibroblasts. *HAS3* expression increased only in OFs after PDGF-BB treatment, and GCs could compensate this stimulating effect. This underscores the multifaceted beneficial effects of GCs in GO.

Li et al. found that, *HYAL1* expression increased 4 h after PDGF-BB treatment, but their 24 h results are constant with our findings, *HYAL1* expression decreased and *HYAL2* remained unchanged [36].

Another mechanism of the direct beneficial effect of GCs may be related to adenosine monophosphate activated protein kinase (AMPK). An appropriate level of energy stores is required for the synthesis of HA in each cell [37]. HA is synthesized utilizing two cytosolic sugar nucleotides: uridine-diphosphate-glucuronic-acid (UDP-GlcUA) and UDP-N-acetylglucosamine (UDP-GlcNAc) as precursors; the availability of these two substrates plays an important role in the regulation of HA synthesis, influencing mRNA expression of HAS enzymes by altering the activity and stability of enzymes required for their synthesis [38]. HAS enzymes do not directly use ATP, but it is required for the production of the precursors; when the ATP:AMP ratio within the cell decreases, AMPK is activated, which inhibits HA synthesis, and restores ATP [39]. GCs increase the phosphorylation of AMPK [40] which can lead to decreasing HA production.

In conclusion GCs, in addition to modulating the immune process, can directly inhibit HA production of orbital fibroblasts. Decreased *HAS* expression and the consequent reduced HA production may be contributors of the beneficial effect of GCs in GO.

## 4. Materials and Methods

### 4.1. Materials

Medium 199 with Earles’ salts, stable glutamine supplement, penicillin/streptomycin, Dulbecco’s phosphate-buffered saline without calcium and magnesium (DPBS) were purchased from Biosera (Nuaille, France). Fetal bovine serum (FBS), TrypLE Express were purchased from Gibco (Thermo Fisher Scientific, Waltham, MA, USA). Methylprednisolone (MP), prednisolone (P), hydrocortisone (HC), dexamethasone (DEX) and dimethyl-sulfoxide (DMSO) were purchased from Sigma Aldrich (St. Louis, MO, USA). A DuoSet Hyaluronan Kit and Recombinant human PDGF-BB were purchased from R&D Systems (Bio-Techne, Minneapolis, MN, USA). Cell proliferation ELISA BrdU (colorimetric) kits were purchased from Roche (F. Hoffmann-La Roche Ltd., Basel, Switzerland). TRI reagent solution was purchased from Molecular Research Center, Inc. (Cincinnati, OH, USA). A High-Capacity cDNA Reverse Transcription Kit and TaqMan Gene Expression Assays were purchased from Applied Biosystems (Thermo Fisher Scientific, Waltham, MA, USA).

### 4.2. Tissue Samples and Cell Cultures

Tissue samples from patients who underwent orbital surgery and abdominal hernia patients who were operated on at the Department of Ophthalmology and the Department of Surgery, respectively, were used. Written consent has been obtained from each patient after full explanation of the purpose and nature of all procedures used.

Fibroblast cultures were generated according to the method previously described [23]. The GO OF cell cultures originated from orbital connective tissue removed during decompression surgery from four patients with inactive GO. Prior to orbital surgery, two patients underwent thyroidectomy, two patients received radioiodine treatment, and all but one patient received thyreostatic drugs during the course of the disease. All patients had received GC therapy and two patients underwent orbital irradiation; at least 12 months had passed since the end of GC treatment and irradiation. Patients had low, normal or suppressed TSH and high–normal thyroid hormone levels within the reference range at the time of surgery. The control OF cultures (NON-GO) originated from connective tissue removed during enucleation surgery from four patients with no history of thyroid disease. Dermal fibroblasts (DF) originated from dermal tissues were obtained during four abdominal hernia surgeries.

Cells were plated in 24-well plates at 15,000 cell/cm^2^, which equals confluent cell density in our model system. After 24 h, cells were synchronized by 24 h serum deprivation. Cells were incubated for an additional 24 h with media containing only vehicle (control) or media containing the various treatments. Supernatants were collected and stored at −20 °C until measurements. Each experiment was performed at least three times and in triplicate. PDGF-BB was dissolved in 4 mM hydrogen chloride (HCl), and its final concentration in the culture media was 10 ng/mL. MP, P, HC and DEX were dissolved in DMSO. The final concentration of DMSO in the media was 0.2%.

### 4.3. Cell Proliferation Assay

A cell proliferation ELISA using BrdU colorimetric assay was performed in 96-well plates according to the manufacturer’s instructions. Briefly, the 5-bromo-2′-deoxyuridine (BrdU) solution was added to the cell cultures and incubated for the last 2 h of the treatments. Then cells were fixed and peroxidase-conjugated, anti-BrdU antibody was added. Finally, 3,3′,5,5′-tetramethylbenzidine substrate was added for 10 min, and after the addition of 2 N H_2_SO_4_, the absorbance was detected at 450 nm (reference wavelength: 620 nm) using a Beckman Coulter, DTX 880 Multimode Detector (Beckman Coulter Inc., Brea, CA, USA).

### 4.4. Quantitation of HA

HA levels in cell culture supernatants were measured using DuoSet Hyaluronan Kit, according to the manufacturer’s instructions. The results were adjusted for the HA content of the FBS used in the media. In all experiments, HA production per confluent cell culture is expressed in nanograms, which was calculated from measured HA concentration in the supernatant and corrected for the amount of culture media.

### 4.5. Real-Time Polymerase Chain Reactions (RT-PCR)

The supernatants were removed, and TRI reagent was used for the isolation of RNA from cells. The purified RNA samples were reverse transcribed by High-Capacity cDNA Reverse Transcription Kit. The TaqMan Gene Expression Assay was used for the detection of the expression of *HAS1*, *HAS2*, *HAS3***,** *HYAL1***,** *HYAL2* and glyceraldehyde 3-phosphate dehydrogenase (*GAPDH*) (assay IDs: HAS1–Hs00987418_m1, HAS2–Hs00193435_m1, HAS3–Hs00193436, HYAL1-Hs00201046_m1, HYAL2-Hs01117343_g1 and GAPDH– Hs02758991_g1). The reactions were performed by the Real-time PCR System (BioRad, Hercules, CA, USA). The results were normalized to *GAPDH* mRNA levels by the ΔCT method.

### 4.6. Statistical Analysis

Statistical analysis was performed using the STATISTICA 14.0 software (TIBCO Software Inc., Palo Alto, CA, USA). Data are presented as mean ± standard error of mean (SEM). Differences among groups were evaluated by a repeated measures ANOVA followed by Dunnett’s test. Statistical significance was set at the 5% level (*p* < 0.05).

## Data Availability

Not applicable.

## References

[1] Bahn R.S. (2010). Graves’ ophthalmopathy. N. Engl. J. Med..

[2] Mishra S., Maurya V.K., Kumar S., Ankita, Kaur A., Saxena S.K. (2020). Clinical Management and Therapeutic Strategies for the Thyroid-Associated Ophthalmopathy: Current and Future Perspectives. Curr. Eye Res..

[3] Wiersinga W.M. (2012). Quality of life in Graves’ ophthalmopathy. Best Pract. Res. Clin. Endocrinol. Metab..

[4] Bartalena L., Kahaly G.J., Baldeschi L., Dayan C.M., Eckstein A., Marcocci C., Marinò M., Vaidya B., Wiersinga W.M. (2021). The 2021 European Group on Graves’ orbitopathy (EUGOGO) clinical practice guidelines for the medical management of Graves’ orbitopathy. Eur. J. Endocrinol..

[5] Längericht J., Krämer I., Kahaly G.J. (2020). Glucocorticoids in Graves’ orbitopathy: Mechanisms of action and clinical application. Ther. Adv. Endocrinol. Metab..

[6] Londzin-Olesik M., Kos-Kudla B., Karpe J., Nowak A., Nowak M. (2021). The Effect of Immunosuppression on Selected Antioxidant Parameters in Patients with Graves’ Disease with Active Thyroid-Associated Orbitopathy. Exp. Clin. Endocrinol. Diabetes.

[7] Sackstein R. (1993). Effects of methylprednisolone administration on lymphocyte LECAM-1, CD44, and LFA-1 expression. Implications for steroid-induced lymphopenia. Ann. N. Y. Acad. Sci..

[8] Korducki J.M., Loftus S.J., Bahn R.S. (1992). Stimulation of glycosaminoglycan production in cultured human retroocular fibroblasts. Invest. Ophthalmol. Vis. Sci..

[9] Sisson J.C. (1971). Stimulation of glucose utilization and glycosaminoglycans production by fibroblasts derived from retrobulbar tissue. Exp. Eye Res..

[10] Scott J.E. (1992). Supramolecular organization of extracellular matrix glycosaminoglycans, in vitro and in the tissues. FASEB J..

[11] Hascall V.C., Wang A., Tammi M., Oikari S., Tammi R., Passi A., Vigetti D., Hanson R.W., Hart G.W. (2014). The dynamic metabolism of hyaluronan regulates the cytosolic concentration of UDP-GlcNAc. Matrix. Biol..

[12] van Steensel L., Hooijkaas H., Paridaens D., van den Bosch W.A., Kuijpers R.W., Drexhage H.A., van Hagen P.M., Dik W.A. (2012). PDGF enhances orbital fibroblast responses to TSHR stimulating autoantibodies in Graves’ ophthalmopathy patients. J. Clin. Endocrinol. Metab..

[13] Bahn R.S. (2003). Pathophysiology of Graves’ ophthalmopathy: The cycle of disease. J. Clin. Endocrinol. Metab..

[14] Bednarczuk T., Gopinath B., Ploski R., Wall J.R. (2007). Susceptibility genes in Graves’ ophthalmopathy: Searching for a needle in a haystack?. Clin. Endocrinol..

[15] Gebhardt C., Averbeck M., Diedenhofen N., Willenberg A., Anderegg U., Sleeman J.P., Simon J.C. (2010). Dermal hyaluronan is rapidly reduced by topical treatment with glucocorticoids. J. Invest. Dermatol..

[16] Smith T.J. (1984). Dexamethasone regulation of glycosaminoglycan synthesis in cultured human skin fibroblasts. Similar effects of glucocorticoid and thyroid hormones. J. Clin. Invest..

[17] Deshpande M., Papp S., Schaffer L., Pouyani T. (2015). Hydrocortisone and triiodothyronine regulate hyaluronate synthesis in a tissue-engineered human dermal equivalent through independent pathways. J. Biosci. Bioeng..

[18] Stuhlmeier K.M., Pollaschek C. (2004). Glucocorticoids inhibit induced and non-induced mRNA accumulation of genes encoding hyaluronan synthases (HAS): Hydrocortisone inhibits HAS1 activation by blocking the p38 mitogen-activated protein kinase signalling pathway. Rheumatology.

[19] Gianoukakis A.G., Jennings T.A., King C.S., Sheehan C.E., Hoa N., Heldin P., Smith T.J. (2007). Hyaluronan accumulation in thyroid tissue: Evidence for contributions from epithelial cells and fibroblasts. Endocrinology.

[20] Smith T.J., Bahn R.S., Gorman C.A. (1989). Hormonal regulation of hyaluronate synthesis in cultured human fibroblasts: Evidence for differences between retroocular and dermal fibroblasts. J. Clin. Endocrinol Metab..

[21] Tuuminen R., Syrjälä S., Krebs R., Arnaudova R., Rouvinen E., Nykänen A.I., Lemström K.B. (2013). Combined donor simvastatin and methylprednisolone treatment prevents ischemia-reperfusion injury in rat cardiac allografts through vasculoprotection and immunomodulation. Transplantation.

[22] Kaback L.A., Smith T.J. (1999). Expression of hyaluronan synthase messenger ribonucleic acids and their induction by interleukin-1beta in human orbital fibroblasts: Potential insight into the molecular pathogenesis of thyroid-associated ophthalmopathy. J. Clin. Endocrinol. Metab..

[23] Galgoczi E., Jeney F., Gazdag A., Erdei A., Katko M., Nagy D.M., Ujhelyi B., Steiber Z., Gyory F., Berta E. (2016). Cell density-dependent stimulation of PAI-1 and hyaluronan synthesis by TGF-β in orbital fibroblasts. J. Endocrinol..

[24] Heldin P., Lin C.Y., Kolliopoulos C., Chen Y.H., Skandalis S.S. (2019). Regulation of hyaluronan biosynthesis and clinical impact of excessive hyaluronan production. Matrix Biol..

[25] Papakonstantinou E., Klagas I., Karakiulakis G., Hostettler K., S’ng C.T., Kotoula V., Savic S., Tamm M., Roth M. (2012). Steroids and β2-agonists regulate hyaluronan metabolism in asthmatic airway smooth muscle cells. Am. J. Respir. Cell. Mol. Biol..

[26] Puissant E., Gilis F., Dogné S., Flamion B., Jadot M., Boonen M. (2014). Subcellular trafficking and activity of Hyal-1 and its processed forms in murine macrophages. Traffic.

[27] Tobisawa Y., Fujita N., Yamamoto H., Ohyama C., Irie F., Yamaguchi Y. (2021). The cell surface hyaluronidase TMEM2 is essential for systemic hyaluronan catabolism and turnover. J. Biol. Chem..

[28] Shiozawa J., de Vega S., Cilek M.Z., Yoshinaga C., Nakamura T., Kasamatsu S., Yoshida H., Kaneko H., Ishijima M., Kaneko K. (2020). Implication of HYBID (Hyaluronan-Binding Protein Involved in Hyaluronan Depolymerization) in Hyaluronan Degradation by Synovial Fibroblasts in Patients with Knee Osteoarthritis. Am. J. Pathol..

[29] Spataro S., Guerra C., Cavalli A., Sgrignani J., Sleeman J., Poulain L., Boland A., Scapozza L., Moll S., Prunotto M. (2022). CEMIP (HYBID, KIAA1199): Structure, function and expression in health and disease. FEBS J..

[30] Virakul S., Heutz J.W., Dalm V.A., Peeters R.P., Paridaens D., van den Bosch W.A., Hirankarn N., van Hagen P.M., Dik W.A. (2016). Basic FGF and PDGF-BB synergistically stimulate hyaluronan and IL-6 production by orbital fibroblasts. Mol. Cell Endocrinol..

[31] DeGrendele H.C., Estess P., Siegelman M.H. (1997). Requirement for CD44 in activated T cell extravasation into an inflammatory site. Science.

[32] Peng Y., Lv S., Li Y., Zhu J., Chen S., Zhen G., Cao X., Wu S., Crane J.L. (2020). Glucocorticoids Disrupt Skeletal Angiogenesis Through Transrepression of NF-κB-Mediated Preosteoclast Pdgfb Transcription in Young Mice. J. Bone. Miner. Res..

[33] van Steensel L., Paridaens D., Dingjan G.M., van Daele P.L., van Hagen P.M., Kuijpers R.W., van den Bosch W.A., Drexhage H.A., Hooijkaas H., Dik W.A. (2010). Platelet-derived growth factor-BB: A stimulus for cytokine production by orbital fibroblasts in Graves’ ophthalmopathy. Invest. Ophthalmol. Vis. Sci..

[34] Rhen T., Cidlowski J.A. (2005). Antiinflammatory action of glucocorticoids--new mechanisms for old drugs. N. Engl. J. Med..

[35] Itano N., Kimata K. (2002). Mammalian hyaluronan synthases. IUBMB Life.

[36] Li L., Asteriou T., Bernert B., Heldin C.H., Heldin P. (2007). Growth factor regulation of hyaluronan synthesis and degradation in human dermal fibroblasts: Importance of hyaluronan for the mitogenic response of PDGF-BB. Biochem. J..

[37] Viola M., Karousou E., D’Angelo M.L., Caon I., De Luca G., Passi A., Vigetti D. (2015). Regulated Hyaluronan Synthesis by Vascular Cells. Int. J. Cell Biol..

[38] Vigetti D., Karousou E., Viola M., Deleonibus S., De Luca G., Passi A. (2014). Hyaluronan: Biosynthesis and signaling. Biochim Biophys. Acta..

[39] Vigetti D., Clerici M., Deleonibus S., Karousou E., Viola M., Moretto P., Heldin P., Hascall V.C., De Luca G., Passi A. (2011). Hyaluronan synthesis is inhibited by adenosine monophosphate-activated protein kinase through the regulation of HAS2 activity in human aortic smooth muscle cells. J. Biol. Chem..

[40] Puthanveetil P., Rodrigues B. (2013). Glucocorticoid excess induces accumulation of cardiac glycogen and triglyceride: Suggested role for AMPK. Curr. Pharm. Des..

